# Supplementary material for: Selection and spatial arrangement of building materials during the construction of nest turrets by grass-cutting ants
Source: R Soc Open Sci. 2020 Oct 14;7(10):201312. doi: 10.1098/rsos.201312 (PMC7657914; doi:10.1098/rsos.201312)
Supplement: Supplementary material [file rsos201312supp1.pdf]

SUPPLEMENTARY MATERIAL

Römer, Cosarinsky and Roces. Selection and spatial arrangement of building materials during the construction of nest turrets by grass-cutting ants

Table S1. Statistics for Figure 6 of the main text. Statistical analysis of choices between thick and thin sticks (G-test for the goodness of fit to the ration 1:1) as depending on the stick ordinal number, in both the initial and the subsequent building phase. Statistically significant P-values are marked in red.

Stick ordinal number	G-value	Initial phase P-value	G-value	Subsequent phase P-value
1st stick	3.985	0.046	9.765	0.0018
2nd stick	2.379	0.123	4.439	0.035
3rd stick	1.202	0.273	11.252	0.0008
4th stick	0.223	0.637	7.925	0.0049
5th stick	0.059	0.808	9.751	0.0018
6th stick	0.059	0.808	8.547	0.0035
7th stick	0.604	0.437	2.093	0.148
8th stick	0.604	0.437	5.062	0.024
9th stick	0	1	0.143	0.705
10th stick	0.287	0.592	2.911	0.088
11th stick	1.974	0.16	8.318	0.004
12th stick	2.358	0.125		
13th stick	0.403	0.526		
14th stick	0.403	0.526		
15th stick	1.646	0.2		
16th stick	2.911	0.088		
17th stick	8.318	0.0039		
18th stick	8.318	0.0039		
19th stick	2.911	0.088		
20th stick	0.201	0.654		
21st stick	6.931	0.0085		
22nd stick	1.927	0.165		
23rd stick	1.927	0.165		
24th stick	6.931	0.0085		
25th stick	6.931	0.0085		
26th stick	6.931	0.0085		
27th stick	6.931	0.0085		
28th stick	6.931	0.0085		

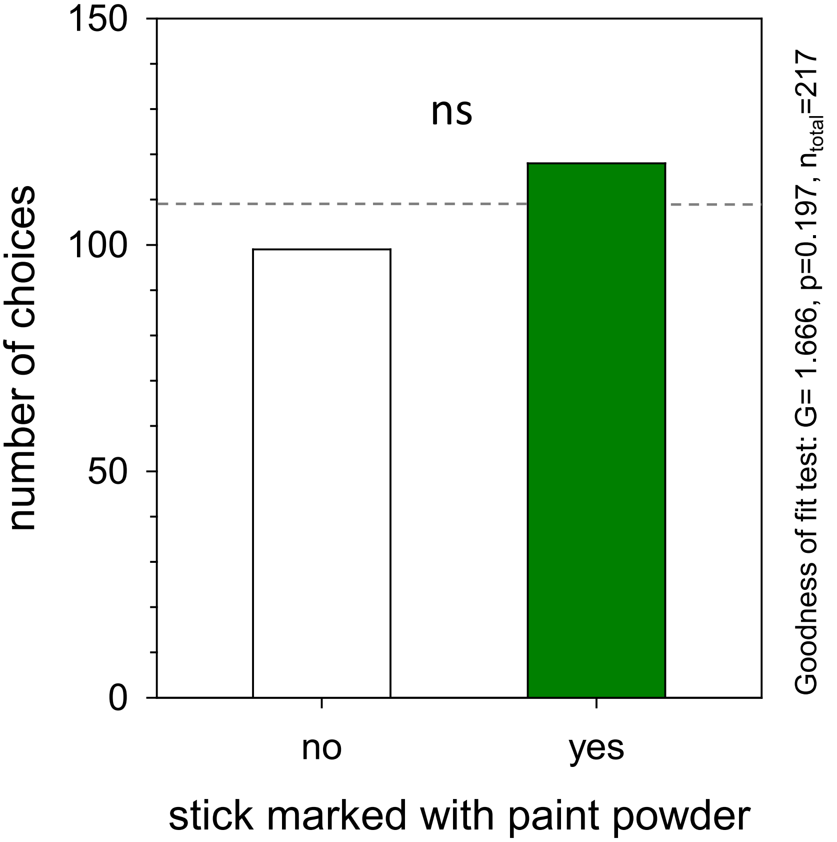

Figures S1: Choice between unmarked and paint-marked thin sticks; dashed line marks the expected 1:1 distribution of choices, ns: $p>0.05$

Choices were tested in the laboratory at the University of Würzburg with the grass-cutting ant *Atta vollenweideri*. Eleven experiments were performed, in which workers could choose between paper strips offered in a pile, either unmarked (white) or coloured with green paint powder. Only the first 20 choices of each experiment were counted. Some choices were deemed invalid, as the ants had picked up both an uncoloured and coloured stick at the same time because they accidentally stuck together. The data of these experiments can be found in the raw data file.
